# Progress in sanitation among poor households in Kenya: evidence from demographic and health surveys

**DOI:** 10.1186/s12889-019-6459-0

**Published:** 2019-01-31

**Authors:** John Njuguna

**Affiliations:** Mukurwe-ini sub-County Public Health Office, P.O. Box 112- 10103, Mukurweini, Kenya

**Keywords:** Kenya, Open defecation, Poor households

## Abstract

**Background:**

An estimated 14% of Kenyans practice open defecation. Poverty has been associated with open defecation. Kenya aims to achieve 100% open defecation free status by 2030 in line with sustainable development goal number 6. Using data from 3 national household surveys, this study sought to explore progress made in attaining this at the household level with a focus on poor households.

**Methods:**

Kenya demographic and health survey for 2003, 2008 and 2014 respectively were analysed. Descriptive analysis and bivariate logistic regression was done with open defecation status as the dependent variable. Independent variables were poverty status, place of residence, region where household was located, absence of farm animals, gender and educational level of household head.

**Results:**

The most common sanitation method nationally is a pit latrine without a slab. This ranged from 35.9–37.9%. Open defecation was 16.2, 12.1 and 9.9% in 2003, 2008 and 2014 respectively. Among households practicing open defecation, 81.8, 86 and 96% were classified as poor in 2003, 2008 and 2014 respectively. Poverty, educational level of household head and residing in a rural area were the most significant predictors of open defecation. Odds ratio for poverty was 9.4 (7–12.6 95% CI), 9.4(6.6–13.5 95% CI) and 29.2 (23.3–36.8 95% CI) for 2003, 2008 and 2014 respectively. The majority of richest households transitioned from using a pit latrine with a slab in 2003 to using a flush toilet connected to a sewer in 2008 and 2014. The majority of richer households transitioned from using a pit latrine without a slab in 2003 and 2008 to using a pit latrine with a slab in 2014. The majority of middle and poorer households stagnated at using a pit latrine without a slab across the similar period. The poorest households stagnated at the open defecation stage.

**Conclusion:**

The burden of open defecation has increased among poor households, more so among the poorest. This may be attributed to non-poor households exiting the open defecation stage at a higher rate compared to poor households. Poor households may need to be targeted more if Kenya is to attain open defecation free status by 2030.

## Background

Unsafe water, sanitation and hand washing is the leading risk factor in Kenya estimated to account for 6250 age-standardised disability adjusted life years (DALY) per 100,000 in 2016. Diarrhoeal diseases accounted for 244.2 years lost to disability (YLD) and 5689.9 years of life lost (YLL) per 100,000 in 2016 [1]. Sanitation is a key intervention in prevention of diarrhoeal diseases. This is because it enables safe containment of human excreta either for disposal on site e.g. in latrines or for disposal offsite e.g. in flush toilets connected to a sewer line. A recent review found that sanitation interventions lower the risk of diarrhoea morbidity by 25%, with evidence of further reduction by 45% when sanitation coverage of above 75% is attained. Interventions promoting hand washing by soap reduce diarrhoea risk by 30% [2]. The aim of sustainable development goal (SDG) number 6 is to ensure availability and sustainable management of water and sanitation for all. A key target of this goal is to achieve access to adequate and equitable hygiene for all and end open defecation, paying attention to the needs of women and girls and those in vulnerable situations eradicate open defecation by the year 2030 [3].

The WHO/UNICEF Joint Monitoring Programme for Water Supply and Sanitation (JMP) has come up with a new sanitation ladder to track progress towards SDG 6 [4]. At the bottom of the ladder is open defecation. This is disposal of human faeces in fields, forests, bushes, and open bodies of water, beaches and other open spaces or with solid waste. Next is unimproved sanitation which is the use of pit latrines without a slab or hanging latrines or bucket latrines. Next on the ladder is limited sanitation. This denotes use of improved facilities shared between two or more households. Improved facilities are those which hygienically separate excreta from human contact. These include various forms of flush toilets, pit latrines with slab and composting toilets. Next is basic sanitation which denotes use of improved facilities which are not shared with other households. At the top of the ladder is safely managed sanitation. This denotes use of non-shared improved facilities where excreta is safely disposed on site or treated off site. For countries to progress up the sanitation ladder, it is imperative that they ensure no household is at the open defecation stage. Open defecation is a risk factor for diarrhoeal diseases including cholera, soil transmitted helminthes [5], and environmental enteropathy [6] which leads to stunting in children. Globally open defecation rates have declined steadily from 1.23 billion to 892 million, an average decrease of 22 million a year [4]. All regions have recorded a decline in open defecation except sub-Saharan Africa and Oceania. In sub-Saharan Africa, open defecation rates increased from 204 to 220 million [4]. Kenya is estimated to have a national open defecation rate of 14% [7, 8]. Though there is wide disparity with some counties like Turkana, Wajir and Samburu having rates of over 70% [7, 8]. Kenya’s policy on sanitation aims to achieve and sustain open defecation free (ODF) status in the entire country by 2030.

Poverty is a determinant of ill health. Poor people may be predisposed to infectious diseases as they tend to live in more polluted environments characterized by lack of clean water and adequate sanitation [9]. In Kenya, poverty has been shown to be associated with open defecation [7, 8]. Poor households which have exited the open defecation stage are more likely to slip back to the open defecation stage. This is because most of them construct simple rudimentary latrines which fill up quickly and are prone to collapse e.g. when subjected to heavy rains or floods. One study found that not being in the richest quintile was significantly associated with slippage occurrence [10].

Poverty is not static and people enter and others exit [11]. Kenya’s rural economy is largely agro-based and subject to the vagaries of weather. When the weather is favourable, farmers harvest bumper harvests and their animals increase. This may cause them to exit poverty. If the reverse occurs, they may slip back into poverty. This is called transient poverty. On the other hand, there are households that don’t exit poverty and these are termed as static. A study done among households in Kenya between 2000 and 2007, indicated that 20% of households exited poverty, 7% descended into poverty, 26.6% were consistently non-poor and 14.4% were chronically poor (i.e. poor every period) [11]. Latest data show that 8.6% of Kenyans live in hardcore poverty [12]. These are households or individuals whose monthly adult equivalent-total consumption expenditure per person is less than 20 US Dollars in rural and peri-urban areas and less than 25 US Dollars in core urban areas. A significant number of poorest households may fall in this category. These may comprise of people living with disabilities, internally displaced persons e.g. due to floods, child-headed households, the landless and the elderly with no one to care for [8]. Slippage to open defecation may be attributed to transient poverty, though this paper is not intended to add any knowledge to this phenomenon. This study sought to explore progress made in sanitation at the household level with a focus on eradication of open defecation among poor households.

## Methods

The study analysed available Kenya’s demographic and health survey data for 2003, 2008 and 2014 respectively. These datasets were obtained from Demographic and Health Surveys (the DHS Program) [13]. These provide information to help monitor the population and health status in Kenya and are nationally representative household surveys. [14]. Number of households interviewed was 40,300; 9936 and 8561 respectively for the surveys in 2014, 2008 and 2003 respectively [14]. Data derived from the household questionnaire was analysed. The household questionnaire collects among other characteristics of the households dwelling unit e.g. source of water, type of toilet facilities, materials used for constructing the floor and roof, and ownership of various durable goods.

## Analysis

The dependent variable was percentage open defecation among households. This was derived from the question on the type of sanitation a household has. Missing values were removed and the responses recoded into a binary variable, namely open defecation and open defecation free using generate and replace commands in Stata [15]. Open defecation was the response that a household has no facility or it uses the bush or field. Open defecation free was all the other responses ranging from flush toilets, pit latrines and hanging toilets. The independent variables were wealth status of household, gender of household head, educational level of household head, place of residence of household i.e. whether urban or rural, district or county where household was located and absence of farm animals in the household. The DHS wealth index categorizes households into 5 wealth quintiles. The poorest and poorer households were recoded as poor while middle, richer and richest were as non-poor. Place of residence is normally stated as urban or rural. Surveys done in 2003 and 2008 had data classifying households as either living in large cities, small cities, small towns and countryside. These were analysed. Educational level of household head was recoded into two categories. The first was no education or pre-school. The second comprised of primary, secondary and tertiary levels.

The svyset command was used to account for the sampling methods used. Using the survey data analysis function, binary logistic regression reporting odds ratio was used. Initially each independent variable was entered on its own and odds ratio determined. Those variables found to be significant were entered into the final model with open defecation and odds ratio determined.

## Results

**Fig. 1:**
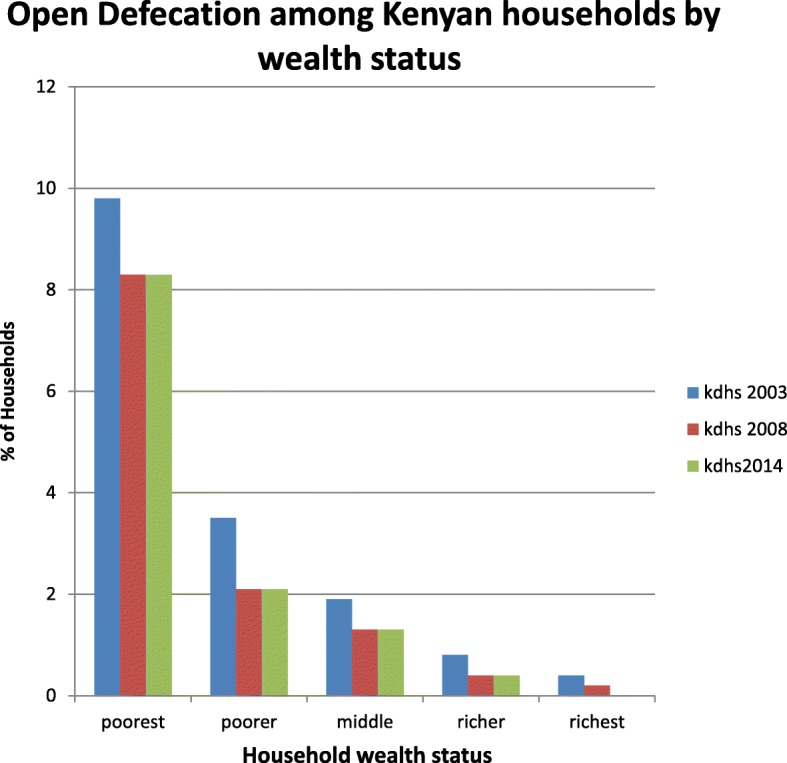
Open Defecation among Kenyan households by wealth status

Nationally, the most common sanitation method is a pit latrine without a slab. This ranges from 35.9 - 37.9% across the surveys. Open defecation was 16.2, 12.1 and 9.9% in 2003, 2008 and 2014 respectively. Among households practicing open defecation, 81.8, 86 and 96% were classified as poor in 2003, 2008 and 2014 respectively. Open defecation declined in all wealth quintiles between 2003 and 2008. Between 2008 and 2014 it stagnated in all quintiles except the richest (Fig. 1).

The most common sanitation method among the poorest households is open defecation (Table 1). Among the poorer and middle groups it’s a pit latrine without a slab. Among the richer group, a pit latrine without a slab was most common in 2003 and 2008 but in 2014 they progressed to a pit latrine with a slab. Among the richest, initially the most common method was a pit latrine with a slab in 2003 before later progressing in 2008 and 2014 to a flush toilet connected to a piped sewer as the most common sanitation method. All the independent variables were significant when compared individually with open defecation status with poverty and educational level of household head having the largest effect (Table 2). The binary logistic regression model showed that poverty and educational level of household head were significant predictors of open defecation. The odds ratio of a poor household practicing open defecation compared to a non-poor household was 9.4 (7–12.6 95% CI) in 2003, 9.4 (6.6–13.5 95% CI) in 2008 and 29.2(23.3–36.8 95% CI) in 2014 (Table 3). The odds of a household whose head has no education or attended school upto to pre-school to practice OD ranged between 3.9–4.3 across the survey period.

**Table 1:** Most common sanitation method as per wealth status ranking (%)

KDHS	Poorest	Poorer	Middle	Richer	Richest
2003	Open defecation (60.1%)	Pit latrine without slab (79.5%)	Pit latrine without slab (83.1%)	Pit latrine without slab (79.4%)	Pit latrine withwithout slab (41.4%)
2008	Open defecation (50.6%)	Pit latrine without slab (64.8%)	Pit latrine without slab (56%)	Pit latrine without slab (35.5%)	Flush toilet to piped sewer (33.1%)
2014	Open defecation (49.6%)	Pit latrine without slab (63.3%)	Pit latrine without slab (53.7%)	Pit latrine with slab (30.3%)	Flush toilet to piped sewer (30%)

**Table 2:** Odds Ratio of Binary factors on Open Defecation among Households

Factor	KDHS 2003	KDHS 2008	KDHS 2014
Gender of household head	0.9 (0.7–1.1)	0.7 (0.6–0.9)	1.6 (1.5–1.8)
Poverty status of household	13.3 (10.2–17.4)	16.9 (11.5–24.8)	58.8 (46.3–74.6)
Absence of farm animals	–	0.4 (0.3–0.6)	0.4 (0.3–0.5)
Educational level of household head	6.0 (4.8–7.4)	6.7 (5.3–8.5)	9.7 (8.5–11.1)
Living in an urban area	0.15 (0.08–0.27)	0.05 (0.02–0.11)	0.08 (0.06–0.1)

**Table 3:** Bivariate logistic regression of Binary variables influencing Open Defecation

Factor	Odds Ratio	Linearized standard error	t	P > |t|	95% CI
Poverty status of household					
KDHS 2003	9.4	1.4	15.1	0.000	7–12.6
KDHS 2008	9.4	1.7	12.3	0.000	6.6–13.5
KDHS 2014	29.2	3.4	28.9	0.000	23.3–36.8
Lives in an urban area					
KDHS 2003	0.6	0.17	−1.8	0.077	0.35–1.1
KDHS 2008	*0.2*	0.09	−3.7	0.000	0.09–0.5
KDHS 2014	0.31	0.04	−9.2	0.000	0.24–0.4
Gender of household head					
KDHS 2003	1.5	0.17	3.3	0.001	1.2–1.8
KDHS 2008	1.3	0.2	2.0	0.04	1–17
KDHS 2014	1.1	0.1	1.6	0.1	1–1.2
Absence of farm animals					
KDHS 2003	n/a				
KDHS 2008	0.9	0.1	−0.8	0.4	0.7–1.2
KDHS 2014	0.7	0.1	−3.8	0.000	0.6–0.9
Educational level of household head					
KDHS 2003	4.3	0.5	11.6	0.000	3.4–5.5
KDHS 2008	4.2	0.6	10.5	0.000	3.2–5.5
KDHS 2014	3.9	0.3	17.7	0.000	3.3–4.5
Region of household(District/County)					
KDHS 2003	1.0	0.6	0.03	0.98	1–1.01
KDHS 2008	1.0	0.03	−0.3	0.8	0.9–1.1
KDHS 2014	1.0	0.003	2.9	0.004	1–1.01

The odds ratio of an urban household to practice OD compared to a rural one ranged between 0.2–0.6 across the survey period. Open defecation among urban households was 0.9, 0.2 and 0.6% for 2003, 2008 and 2014 respectively. In rural households it was 15.3, 11.8 and 9.3% for a similar period. Surveys done in 2003 and 2008 had data on households living in large cities, small cities, small towns and countryside. In 2003, OD was 2.7, 0.7, 6.9 and 20.4% among these four categories. In 2008 it was 0.8, 0, 1.1 and 16% respectively. When all households practicing OD were considered in 2003, 2.2, 0.2, 3.2 and 94.4% were located in large cities, small cities, small towns and the countryside respectively. In 2008, the figures were 0.8, 0, 1.1 and 98.1% respectively. Gender of household head was significant for 2003 and 2008 whereas absence of farm animals was significant in 2014. The region where a household was located was only significant in 2014.

## Discussion

Across the 11 year period open defecation declined nationally from 16.2 to 9.9% with the most common sanitation method being a pit latrine without slab. This is classified as unimproved sanitation. Open defecation declined from 60.1 to 49.6% among poorest households across the 11 year period. This roughly translates to a 1% decline annually. At this current rate of decline, open defecation may not be eliminated among poor households by 2030. The poorer and middle households had a pit latrine without slab as their most common sanitation method. This is classified as unimproved sanitation. The richer households had a pit latrine with a slab as their most common sanitation method. This is classified as improved sanitation. The richest households had a flush toilet connected to a piped sewer as their most common sanitation method. This is classified as improved sanitation if shared among households. If not shared, then it is safely managed sanitation. This indicates that the poorest are at the bottom of the sanitation ladder and the richest are at the top rungs. In between are the poorer, middle and richer households.

Poverty levels, level of education of household head and place of residence were significant predictors of open defecation. The odds of a poor household to practice open defecation was 9.4 for both 2003 and 2008. In 2014, it increased by more than threefold to 29.4. Between 2008 and 2014, OD stagnated among the poorest, poorer, middle and richer wealth quintiles (Fig. 1). These groups had OD levels of 8.3, 2.1, 1.3 and 0.4% respectively. Between 2008 and 2014, OD among the richest declined from 0.2 to 0.002%. This is a decline of 99% and it may have led to the three fold increase in odds of a poor household to practice OD compared to a non-poor household. The WHO/UNICEF Joint Monitoring Programme for Water Supply and Sanitation (JMP) uses a customized wealth index which excludes water and sanitation variables. It shows significant differences in coverage of basic water, hygiene and sanitation across wealth quintiles. The gaps between the wealth quintiles are larger for sanitation than for hygiene or drinking water. The WHO/UNICEF JMP indicates that there are inequities in open defecation in Kenya with 47% of poorest households practicing open defecation compared to 0% of richest households [4]. This study shows that 49.6% of poorest households were practicing open defecation in 2014 compared to 0.002% of richest households. It is estimated that at current rates of reduction, open defecation will not be eliminated among the poorest in rural areas by 2030 [4]. This study shows that open defecation has increasingly been confined to poor households across the survey periods. A study on access to environmental health assets in 41 low and middle income countries found disparities in access between the richest and poorest quintiles. Access to environmental health assets was very low among the poorest and the disparities were greatest for improved sanitation and electricity [16]. Open defecation among Nigerian households was influenced by wealth status, place of residence, geo-political region, ethnicity and household head’s level of education [17].

Poor households may lack a latrine due to a number of reasons. One is that they may be unable to afford one. A study in rural Malawi found that households with no latrines lacked money to construct one. These households were also socially vulnerable; less educated, and often had impaired mental health [18]. In Tanzania, households practicing open defecation cited inability to pay for sanitation infrastructure as a reason for practicing open defecation [19]. In Ethiopia, household income was a determinant of latrine availability. Latrine availability increased two fold in households with an annual income of US Dollars 300 or more per year compared to households with less than US Dollars 300 per year [20].

Secondly, sanitation is poorly funded in Kenya. Kenya is a signatory of the Ngor declaration of 2016 in which it committed itself to focus on the poorest, most marginalized and unserved with the aim of progressively eliminating inequalities as well as eliminating open defecation by 2030 [21]. Towards this end, it committed to invest 0.5% of its gross domestic product (GDP) on sanitation. Currently it has invested 0.2% of its GDP. Sanitation is not given priority and this makes elimination of open defecation difficult [8].

Thirdly, existing sanitation programs may not be pro-poor. This means that the poor may be unserved or underserved by existing sanitation programs. An example is people living with disabilities e.g. the blind, deaf and mentally challenged. These are often overlooked during the design and implementation of sanitation programs e.g. there are no information, communication and education materials in Braille to cater for the blind. Some may be hidden by their families. The already constructed sanitation facilities may not be user-friendly for them and they may resort to open defecation [22]. Most households practicing open defecation are predominantly located in rural areas. One approach widely implemented to eliminate open defecation in rural areas is the non-subsidy based community-led total sanitation approach. It has been argued that the least able or vulnerable groups may need some support to eliminate open defecation [23]. An example may be poor people and people living in areas with hydro-geological conditions which make it difficult to construct latrines e.g. high water table and weak soils prone to collapse. This makes the cost of constructing a simple latrine out of reach for many as the pit has to be lined to avert collapse. When they manage to construct a simple latrine, it’s prone to fill up quickly or collapse. This may result in them slipping back to the OD stage. Slippage to OD or having poorly built or dirty latrines has been associated with poor or most vulnerable communities [22]. A cluster randomized trial in rural Bangladesh aimed at improving sanitation assigned communities to motivation and information; subsidies and a supply-side market access intervention. Subsidies to the majority of landless poor increased latrine ownership and also reduced open defecation [24]. A meta-analysis on impact of sanitation interventions on latrine coverage and use found that latrine subsidy with provision of interventions that incorporated an education component attained a 17% increase compared to 12% for community-led-total sanitation [25]. Support may include provision of technical support and external support e.g. conditional cash transfer and vouchers.

This study showed that households whose head did not have any formal education or only went up to pre-school were four times more likely to practice OD compared to a household whose head had an educational level of primary school and above. In Ethiopia, a study found that households whose head had a level of education of primary school and above were twice likely to utilize a latrine compared to households whose head was illiterate [26]. In Nigeria, OD among households has been shown to be influenced by the household head’s level of education [17]. The more educated a household head, the more likely they are to understand the importance of sanitation facilities. They are also more likely to earn more compared to their semi- illiterate counterparts and may be in a better position to afford a sanitation facility.

Open defecation is low in urban areas compared to rural areas. A study has shown that access to environmental health assets is higher in urban areas compared to rural areas except for bed nets. [16]. Open defecation was 0.6% in urban areas compared to 9.3% in rural areas in 2014. In Nigeria OD was 8% in urban areas compared to 24% in rural areas in 2013 [17]. There are a number of reasons for this. Poverty levels tend to be lower in urban areas compared to rural areas. This means majority of urban households can afford sanitation facilities compared to their rural counterparts. Urban areas tend to have a high population density making it difficult to practice open defecation due to limited privacy compared to sparsely populated rural areas. Enforcement of sanitation related laws requiring households to have sanitation facilities is relatively high in urban areas compared to rural areas. In Kenya, the Public Health Officers and Technicians enforce this through the Public Health Act. Urban areas especially cities also have capital intensive sanitation projects like sewerage systems. A reduction in open defecation in cities was associated with higher levels of external funding for water supply and sanitation [27]. Nairobi, the capital city of Kenya has an open defecation rate of less than 1%. Despite this, cities prevalence in open defecation is increasing, with an annual increase of 0.3% among 26 cities. A reason for this is that the sanitation improvements are not available to the poorest and marginalized [27]. The poorest quintile in urban areas has been shown to be disadvantaged in terms of access to environmental health assets. This may be the reason why this study showed a decline in open defecation followed by an increase among urban households in Kenya. This study shows that in 2008, OD had declined in large cities, small towns, countryside and had been eliminated in small cities in Kenya. Demographic and health surveys define large cities as either capital cities or cities with a minimum population of one million. Small cities are defined as having a minimum population of 50,000. Other urban areas are classified as small towns and all rural areas classified as countryside [28]. Small cities tend to be better planned and devoid of population pressure due to rural –urban migration compared to large cities. Large cities tend to have a significant proportion of their population living in informal settlements. These are characterized by poor water and sanitation services [29]. Small cities also tend to be better funded and better planned with enforcement of regulations compared to small towns.

## Limitations

Some households may have sanitation facilities and still practice OD due to personal beliefs and customs. This may be intermittent e.g. cultures which don’t allow one to share sanitation facilities with their in laws. When the in laws visit, the head of the household may practice OD. These cannot be captured in DHS surveys. The DHS wealth index is constructed using the principal component analysis method which analyses type of sanitation facility as one of the measures. This may introduce bias and the study was unable to construct its own wealth indices excluding sanitation. In spite of this, studies using wealth indices which excluded sanitation also had similar results with respect to the disparities between the richest and poorest in terms of sanitation ownership [4, 16]. Also DHS data are essentially cross-sectional meaning no causal claims can be made. The logistic regression model used did not account for time.

## Conclusion

A household practicing OD in Kenya is likely to be poor, based in a rural area and having a illiterate/ semi-illiterate head. Poverty was the most significant factor with the odds of a poor household to practice open defecation increasing three fold between 2008 and 2014. Half of poorest households still practice open defecation. Poor households may need to be assisted e.g. through subsidies to acquire their own latrines. This will ensure that they exit the open defecation stage of the sanitation ladder and contribute to the attainment of SDG 6 in Kenya.

## Abbreviations

DHS: The Demographic and Health Surveys Program
OD: Open defecation
The WHO/UNICEF JMP: The WHO/UNICEF Joint Monitoring Programme for Water Supply and Sanitation (JMP)
USD: United States Dollars

## References

[1] Achoki T, Miller-Petrie MK, Glenn SD, Kalra N, Lesego A, Gathecha GK, et al. Health disparities across the counties of Kenya and implications for policy makers, 1990–2016: a systematic analysis for the Global Burden of Disease Study 2016. Lancet Glob Health:2018. 10.1016/S2214-109X(18)30472-8. 10.1016/S2214-109X(18)30472-8 PMC6293072 30482677

[2] Wolf J, Hunter PR, Freeman MC, Cumming O, Clasen T, Bartram J (2018). Impact of drinking water, sanitation and hand washing with soap on childhood diarrhoeal disease: updated meta-analysis and meta-regression. Tropical Med Int Health.

[3] United Nations. Transforming our world: The 2030 Agenda for sustainable development 2015 https://sustainabledevelopment.un.org/content/documents/21252030%20Agenda%20for%20Sustainable%20Development%20web.pdf. Accessed 4 Jan 2019..

[4] The WHO/UNICEF Joint Monitoring Programme for Water Supply and Sanitation (JMP). Progress on drinking water, sanitation and hygiene: 2017 Update and SDG baselines. Geneva: World Health Organization (WHO) and the United Nations Children’s Fund (UNICEF); 2017.

[5] Ganguly S, Barkataki S, Karmakar S, Sanga P, Boopathi K, Kanagasabai K (2017). High prevalence of soil transmitted helminth infections among primary school children, Uttar Pradesh, India,2015. Infect Dis Poverty.

[6] Spears D, Ghosh A, Cumming O (2013). Open defecation and childhood stunting in India: an ecological analysis of data from 112 districts. PLoS One.

[7] Republic of Kenya. National ODF Kenya 2020 Campaign Framework 2016/17–2019/20. Nairobi: Ministry of Health; 2015.

[8] Republic of Kenya (2015). Kenya Environmental Sanitation and Hygiene Policy 2016-2030.

[9] Waller LA, Louis TA, Carlin BP (1999). Environmental justice and statistical summaries of differences in exposure distributions. J Expos Anal Environ Epidemiol.

[10] Odagiri M, Muhammad Z, Cronin AA, Gnilo ME, Mardikanto AK, Umam K (2017). Enabling factors for sustanining open defecation-free communities in rural Indonesia: a cross-sectional study. Int J Environ Res Public Health.

[11] Suri T, Tschirley D, Irungu C, Gitau R, Kariuki D (2008). Rural incomes, inequality and poverty dynamics in Kenya. Tegemeo Institute of Agricultural Policy and Development.

[12] Republic of Kenya (2018). Kenya integrated household budget survey: Basic reports.

[13] The Demographic and Health Surveys Program. https://dhsprogram.com/. (2018). Accessed 5 Jul 2018.

[14] Kenya National Bureau of Statistics (KNBS) and ICF International (2015). Kenya demographic and health survey 2014.

[15] StataCorp (2011). Stata Statistical Software: Release 12.

[16] Kaur M, Jeuland MA (2018). Access to environmental health assets across wealth strata: evidence from 41 low- and middle income countries. PLoS One.

[17] Abubakar IR (2018). Exploring the determinants of open defecation in Nigeria using demographic and health survey data. Sci Total Environ.

[18] Slekiene J, Mosler HJ (2018). Characterizing the last latrine nonowners in rural Malawi. Am J Trop Med Hyg.

[19] Sara S, Graham J (2014). Ending open defecation in rural Tanzania: which factors facilitate latrine adoption?. Int J Environ Res Public Health.

[20] Awoke W, Muche S (2013). A cross sectional study: latrine coverage and associated factors among rural communities in the district of Bahir Dar Zuria, Ethiopia. BMC Public Health.

[21] The NGOR declaration on Sanitation and Hygiene.2015 https://www.wsscc.org/resources-feed/the-ngor-declaration-on-sanitation-and-hygiene/ Accessed 29 Aug 2018.

[22] Wilbur J, Jones H (2014). ‘Disability: making CLTS fully inclusive’, Frontiers of CLTS: innovations and insights issue 3.

[23] Myers J, Gnilo M (2017). ‘Supporting the poorest and Most vulnerable in CLTS Programmes’, CLTS knowledge hub learning paper.

[24] Guiteras R, Levinsohn J, Mubarak AM (2015). Sanitation subsidies: Encouraging sanitation investment in the developing world: a cluster-randomized trial. Science.

[25] Gain JV, Sclar GD, Freeman MC, Penakalapati G, Alexander KT, Brooks P (2017). The impact of sanitation interventions on latrine coverage and latrine use: a systematic review and meta-analysis. Int Hyg Environ Health.

[26] Gebremedhin G, Tetemke D, Gebremedhin M, Zelalem H, Syum H, Gerensea H (2018). Factors associated with latrine utilization among model and non-model families in Laelai Maichew Woreda Aksum, Tigray, Ethiopia: comparative community based study. BMC Res Notes.

[27] Hopewell MR, Graham JP (2014). Trends in access to water supply and sanitation in 31 major sub-Saharan African cities: an analysis of DHS data from 2000 to 2012. BMC Public Health.

[28] The Demographic and Health Surveys Program (2018). Standard Recode manual for DHS7.

[29] Kamau N, Njiru H (2018). Water, sanitation and hygiene situation in Kenya’s urban slums. J Health Care Poor Underserved.

